# A Retrospective Study on the Prevalence and Antimicrobial Susceptibility of Gram-Positive Cocci in a Pediatric Department: A Single-Center Report from Egypt

**DOI:** 10.3390/medicina61061089

**Published:** 2025-06-14

**Authors:** Mona Moheyeldin AbdelHalim, Shimaa A. Abdel Salam, Marwa O. Elgendy, Ahmed M. Abdel Hamied, Sultan M. Alshahrani, Ahmed R. N. Ibrahim, Heba Sherif Abdel Aziz

**Affiliations:** 1 Department of Clinical and Chemical Pathology, Faculty of Medicine, Cairo University, Cairo 12613, Egypt; mona.mohiedden@kasralainy.edu.eg; 2 Department of Medical Microbiology and Immunology, Faculty of Medicine, Ain Shams University, Cairo 11566, Egypt; shimaa_ahmed@med.asu.edu.eg; 3 Department of Clinical Pharmacy, Beni-Suef University Hospitals, Faculty of Medicine, Beni-Suef University, Beni-Suef 62521, Egypt; 4 Department of Pediatrics, Faculty of Medicine, Nahda University (NUB), Beni-Suef 62513, Egypt; Ahmed.abdelhamid@nub.edu.eg; 5 Department of Clinical Pharmacy, College of Pharmacy, King Khalid University, Abha 61421, Saudi Arabia; shahrani@kku.edu.sa (S.M.A.); aribrahim@kku.edu.sa (A.R.N.I.); 6 Department of Biochemistry, Faculty of Pharmacy, Minia University, Minia 61511, Egypt

**Keywords:** antimicrobial susceptibility, gram-positive bacteria, pediatric hospital

## Abstract

*Background and Objectives*: The rising prevalence of drug-resistant organisms presents a significant challenge to healthcare, underscoring the importance of implementing effective antimicrobial stewardship programs. The success of these programs depends on access to accurate, evidence-based data reflecting local patterns of antibiotic resistance. This study aims to assess the antimicrobial susceptibility profiles of gram-positive bacteria isolated from pediatric patients in a tertiary care hospital in Egypt. *Materials and Methods*: We carried out a retrospective study over a five-year period, from January 2018 to December 2022, using microbiology laboratory records. Clinical samples included blood, urine, respiratory secretions, pus, wound, cerebrospinal fluid (CSF), and pleural fluid. The analysis focused on the resistance patterns of gram-positive pathogens identified through routine culture procedures. Antimicrobial susceptibility testing was performed using the Kirby–Bauer disc diffusion method, and vancomycin MIC was confirmed using the VITEK 2 system. *Results*: A total of 3223 gram-positive bacterial isolates were identified. *Staphylococcus aureus*, including 82.5% methicillin-resistant strains (MRSA), exhibited high resistance to erythromycin (47.3%) and gentamicin (low potency) (32.1%). Coagulase-negative staphylococci (CoNS) showed the highest erythromycin resistance (up to 88.3%), while *Enterococcus* spp. demonstrated declining susceptibility to vancomycin, levofloxacin, and erythromycin. Across all isolates, vancomycin and gentamicin (high potency) showed the highest overall susceptibility. Resistance to cotrimoxazole and doxycycline declined over the five-year period. *Conclusions*: While a decline in resistance was noted for some agents, persistent resistance to key antibiotics (particularly erythromycin and gentamicin) among MRSA and CoNS remains concerning. These findings underscore the importance of targeted antimicrobial stewardship interventions and continuous surveillance to inform empirical therapy in pediatric settings.

## 1. Introduction

Antimicrobial resistance (AMR) is a serious public health threat, placing a significant burden on both healthcare systems and patients, both in terms of health outcomes and economic costs [1]. If no action is taken to address antimicrobial resistance (AMR), it is projected to cause global economic losses of up to USD 3 trillion each year by 2050, along with an estimated 10 million deaths annually. Over time, the total cost could exceed USD 100 trillion [2]. One of the major challenges in addressing AMR is the declining investment from the private sector in the development of new antimicrobial drugs, which weakens global efforts to tackle this issue [3]. AMR is a worldwide concern that demands international cooperation and urgent action. In Egypt, the misuse of antibiotics is widespread, largely due to the ability of individuals to purchase them over the counter from community pharmacies without a prescription. Although the Egyptian Ministry of Health has introduced regulations restricting the sale of antibiotics without a prescription, non-compliance remains a significant issue [4]. Clinical microbiology laboratory data play a crucial role in identifying bacterial susceptibility to antimicrobial agents, aiding in the monitoring and management of emerging resistance patterns [5]. These data are valuable for selecting appropriate antibiotics for empirical therapy before susceptibility test results are available. However, their use for epidemiological surveillance remains limited. While antimicrobial stewardship programs primarily focus on optimizing antibiotic prescribing practices, they also rely on a thorough understanding of local antibiotic resistance trends [6]. This, in turn, depends on the availability of reliable medical microbiology laboratory resources. Since January 2014, the Medical Group has implemented antimicrobial stewardship (AMS) programs, utilizing various strategies to curb inappropriate antibiotic use, reduce AMR development, lower healthcare-associated infections (HAIs), and decrease overall healthcare costs [1,7].

Several studies have assessed antibiotic susceptibility rates among gram-positive bacteria in Egypt. However, none have provided a comprehensive overview, and comparisons between studies are challenging due to differences in methodologies and study periods. This study aimed to analyze antimicrobial susceptibility patterns of gram-positive pathogens isolated from pediatric patients, using surveillance data collected over a five-year period in a pediatric hospital in Egypt.

## 2. Materials and Methods

### 2.1. The Study Design

A five-year retrospective study was conducted at a pediatric tertiary care hospital in Egypt from January 2018 to December 2022.

Ethical approval was obtained from the Research Ethics Committee, Faculty of Medicine, Cairo University. Microbiological data were retrieved from the Laboratory Data Manager (LDM) system (Soft Computer Consultants, FL, USA), focusing on antimicrobial susceptibility profiles of gram-positive bacteria isolated from routinely submitted clinical specimens.

### 2.2. Microbiological Specimen Processing and Identification

The clinical specimens analyzed included blood, urine, respiratory secretions, wound and pus swabs, cerebrospinal fluid (CSF), and pleural fluid. All samples were processed according to standard operating procedures. Initial isolation was performed using appropriate culture media (Oxoid, Hampshire, UK) and incubation at 35 °C for 24–48 h. Blood cultures were processed using the Bact/ALERT system (bioMérieux, Inc., Salt Lake City, UT, USA).

The blood cultures were incubated for seven to ten days. Initially, positive blood culture bottles were cultivated using blood agar, chocolate agar, and MacConkey agar for 24–48 h at 35–37 °C [8].

The identification of isolated microorganisms involved gram staining, standard confirmatory biochemical tests, and an examination of colony morphology. Hemolytic activity on blood agar was utilized for identifying gram-positive bacteria, followed by further characterization using various biochemical tests, such as the catalase reaction, slide and tube coagulase tests, DNase agar culture, Mannitol salt agar, and bile esculin [8]. No automated or molecular identification systems were employed during the study period; biochemical tests were considered sufficient for identification based on routine practice and resource availability.

### 2.3. Antimicrobial Susceptibility Testing

Antimicrobial susceptibility testing for the bacterial isolates was performed using the Kirby–Bauer disc diffusion method. A standardized 0.5 McFarland inoculum was spread on Mueller Hinton agar (Oxoid, Ltd., Hampshire, UK), and antibiotic discs were applied using an automated dispenser (Oxoid Ltd., Hampshire, UK). After overnight incubation at 35 °C, inhibition zones were interpreted per CLSI guidelines, except for tigecycline, which followed EUCAST standards [9,10,11,12,13,14]. The antibiotic discs used in the testing were regularly provided by Oxoid, Hampshire, UK. Cefoxitin discs (30 µg) were used for MRSA detection, and vancomycin screening agar (brain heart infusion agar (BHI) with 6 μg/mL vancomycin) was used for preliminary detection of Vancomycin-resistant *Enterococci* (VRE) and vancomycin-resistant Staphylococcus, followed by MIC confirmation via the VITEK 2 system (bioMérieux, Marcy-l’Étoile, France).

### 2.4. Quality Control

The sterility of the recently opened medium was verified before its use. To assess the performance of each medium and antimicrobial disc, recommended reference strains, including *E. coli* (ATCC^®^25922), *S. aureus* (ATCC^®^25923), *K. pneumoniae* (ATCC^®^700603), *S. pneumoniae* (ATCC^®^49619), and *P. aeruginosa* (ATCC^®^27853), were employed.

### 2.5. Data Collection and Analysis

The data retrieved from the LDM included only final verified results of pathogens with ≥30 isolates tested that were included against routinely used antimicrobial agents during the routine diagnosis and not for surveillance purposes. The data were further stratified into inpatients and outpatients for further analysis and comparison.

### 2.6. Statistical Methods

The data were entered and coded using IBM Corp.’s statistical software for the social sciences, SPSS v28 (Armonk, NY, USA). Percentages were used to summarize the data, including frequencies (number of cases) and relative frequencies.

Categorical variables were compared using the Chi-square test; Fisher’s exact test was applied when expected frequencies were < 5. A *p* value ≤ 0.05 was considered statistically significant.

## 3. Results

A total of 3223 gram-positive bacterial isolates were identified from pediatric patients between 2018 and 2022.

### 3.1. The Prevalence of Gram-Positive Pathogens in the Pediatric Tertiary Care Hospital over a Period of Five Years

The highest number of isolates was recorded in 2021 (716; 22.2%), followed by 2022 (653; 20.26%). Most of the *S. aureus* was retrieved in 2020 (23.82%). For CONS, most of the isolates were isolated in 2021 (24.78%), while *Enterococci* was mostly isolated in 2018 (24.33%). Regarding the MDROs, MRSA represented 82.5% of all *S. aureus* isolates (716/869). The highest MRSA frequency was observed in 2018 (90.7%), while no vancomycin-resistant *S. aureus* (VRSA) isolates were detected during the study period.

The prevalence and distribution of gram-positive pathogens in different specimens over 5 years is shown in Supplementary Tables S1–S4.

**Figure 1:**
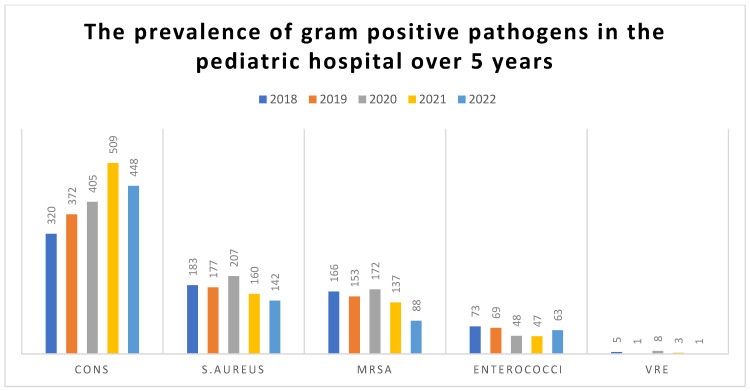
The prevalence of gram-positive Cocci in the pediatric hospital over 5 years.

The frequency of Vancomycin-resistant *Enterococci* (VRE) was rare (18/300; 6%), peaking in 2020 (16.7%). Significant differences in yearly distributions were observed for each pathogen (*p* ≤ 0.001). The distribution of isolates by species over the study period is shown in Table 1 and Table 2 and Figure 1.

**Figure 2:**
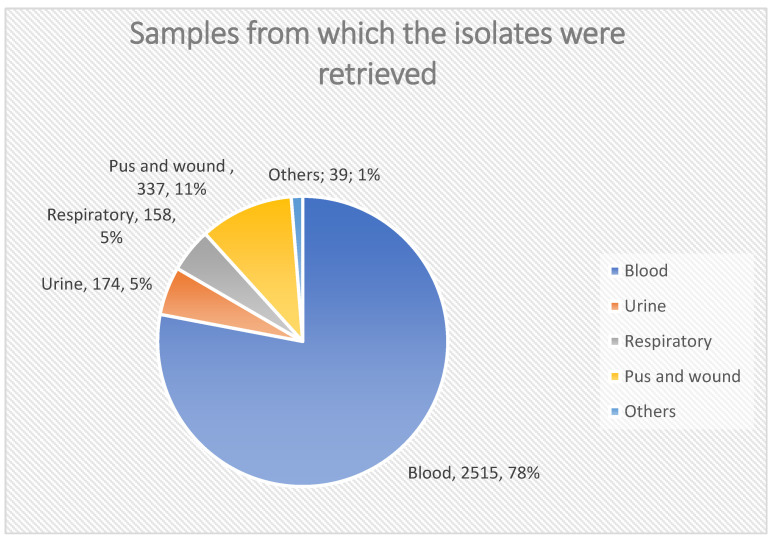
Samples from which the isolates were retrieved.

Most of the gram-positive isolates were retrieved from blood samples (78%), with CoNS being the most common isolate from blood (59.4%). All respiratory samples yielded *Staphylococcus aureus*, with MRSA accounting for 89% (141/158) of these isolates, as shown in Figure 2.

### 3.2. Antimicrobial Susceptibility Profile of All Gram-Positive Pathogens over 5 Years

A study of the antibiotic resistance profiles of various gram-positive organisms was conducted. The results indicate that Gm-positive bacteria over 5 years showed the highest percentage of susceptibility to vancomycin, followed by Clindamycin and levofloxacin. By comparing the susceptibility profile over the five years, it was found that resistance for SXT and high-potency gentamicin antibiotics significantly increased in 2022 compared to previous years, and resistance to levofloxacin was significantly high in 2020 compared to other years, while doxycycline showed high resistance in 2018. Although erythromycin showed low susceptibility over the tested 5 years, there was no statistically significant difference in susceptibility distribution.

The antimicrobial susceptibility profile in various gram-positive organisms is illustrated in Table 3, and a statistically significant variation in the antimicrobial susceptibility of Gm-positive bacteria over the 5 years was found in different antibiotics, as presented in Table 3.

### 3.3. The Antimicrobial Susceptibility of Different Gram-Positive Bacteria

#### 3.3.1. *Staphylococcus aureus*

- Vancomycin maintained 100% susceptibility throughout the study period.
- Resistance to erythromycin ranged from 37.4% to 51.7%, with no significant year-to-year change (*p* = 0.078).
- Cotrimoxazole resistance significantly declined from 24% in 2018 to 20.9% in 2022 (*p* ≤ 0.001).
- Doxycycline susceptibility increased significantly over time (from 39% to 57.4%, *p* ≤ 0.001) (Table 4).

#### 3.3.2. MRSA

- MRSA isolates showed full susceptibility to vancomycin.
- Resistance to gentamicin (low potency) was highly variable, with a notable decline in 2019 (7.6% susceptibility, *p* ≤ 0.001).
- Doxycycline and cotrimoxazole susceptibility improved significantly (Table 5).

#### 3.3.3. Coagulase-Negative *Staphylococci* (CoNS)

- Vancomycin retained 100% efficacy against CoNS isolates.
- Doxycycline and cotrimoxazole resistance decreased significantly.
- Erythromycin resistance remained high (lowest susceptibility of 11.7% in 2022).
- All antibiotics tested for CoNS showed statistically significant differences throughout the tested 5 years (Table 6).

#### 3.3.4. *Enterococcus* Species

- Vancomycin susceptibility declined from 87% in 2018 to 70.7% in 2021, then rose to 86% in 2022 (*p* = 0.001).
- Erythromycin susceptibility dropped from 32% to 7.1% over the five years (*p* = 0.006).
- Doxycycline susceptibility varied, peaking in 2020 (68.2%), then decreasing (Table 7).

## 4. Discussion

The overuse and misuse of antibiotics have significantly contributed to the increasing incidence of antimicrobial resistance (AMR) [15,16,17]. As a result, AMR has been declared a major global health threat by the World Health Organization (WHO) [18]. In developing countries, such as Egypt, poor infection control, over-the-counter antibiotic availability, and inadequate stewardship practices exacerbate the problem. Despite regulatory efforts by the Egyptian Ministry of Health, enforcement remains inconsistent. Reliable and up-to-date hospital-based resistance data are essential for the design of effective local antimicrobial stewardship interventions [3]. To stop this spiraling, out-of-control issue, comprehensive oversight of antibiotic use in developing countries is necessary. Comprehensive antibiotic stewardship in low-income countries is crucial. However, there is a lack of sufficient data on antimicrobial resistance to accurately assess the problem’s scope. Also, information on antibiotic resistance is not enough to precisely assess the problem’s extent. Hospitals are thought to be breeding grounds for recently emerging high-level resistance based on previous research. More research in other countries and medical facilities is therefore encouraged [19].

Several studies have previously evaluated the performance and reported excellent sensitivity and specificity for bacterial identification and resistance patterns. However, these studies were limited, in that the evaluation period was brief (<1 year), they comprised a relatively small sample size (78 to 250 cultures), and the evaluation was conducted prior to implementation with a primary focus in the adult population.

In this study, coagulase-negative staphylococci (CoNS) were the most frequently isolated pathogens, consistent with previous studies conducted in Egypt [18,20,21]. However, variations in prevalence across studies (e.g., the dominance of *S. aureus* in some reports) may reflect differences in patient populations, sample types, and hospital infection control policies.

The high frequency of CoNS, especially from blood samples, may be attributed to frequent device use, suboptimal aseptic techniques, and handling by healthcare workers [22]. A proper interpretation of CoNS isolates is crucial, as they are often contaminants. Misidentification can lead to unnecessary treatment and resistance development [23].

Another study conducted in Egypt revealed that most gram-positive isolates were Staph species, followed by *Enterococci* [24].

In this study, the *Enterococcus* spp. isolates within the 5 years showed decreased susceptibility towards ciprofloxacin, levofloxacin, and erythromycin where susceptibility was 37%, 50%, and 32% in 2018 and decreased along the years and reached 28.7%, 43 %, and 7.1% in 2022 for ciprofloxacin, levofloxacin, and erythromycin, respectively. These trends are comparable to other regional findings [20,21]. Our study recorded a VRE prevalence of 6%, with the highest peak in 2020 (16.7%).

The frequencies of MRSA in 2018, 2019, 2020, 2021, and 2022 were 90.7%, 86.4%, 83%, 85.6%, and 62%, respectively. No isolates of *S. aureus* showed resistance to vancomycin. All CoNS were sensitive to vancomycin within the study period. The frequency of erythromycin resistance to CoNS was high during the whole study period.

In the current study, 82.5% of the superbug isolates were MRSA, and 6% were VRE, which are higher than figures reported in Saudi Arabia (15.9%) and Ethiopia (45.8%) but consistent with several Egyptian studies (~73–90%) [25,26,27]. This variation may reflect differences in infection control protocols, antibiotic usage pressure, and diagnostic criteria.

Another study conducted in Iran [28] concluded a descending rate of MRSA during the study period (from 95.24% to 36.36%), and the authors did not provide any explanation for these results. A different study in Iran reported different rates of MRSA, ranging between 40% and 100% [29,30]. The rate of MRSA is significantly affected by multiple factors, including infection control measures and antimicrobial selection pressure.

Vancomycin maintained excellent activity against *S. aureus* and CoNS (100% susceptibility), affirming its role as a key treatment option. However, the reduced vancomycin susceptibility in *Enterococcus* spp. (as low as 70.7%) is a cause for concern, possibly due to selection pressure and inappropriate empirical use.

In accordance with our results, the higher susceptibility of *Enterococcus* species to vancomycin (85.9%) and (79.7%) was reported in studies conducted in Saudi Arabia [27,31]. Highest susceptibility to vancomycin and linezolid (98.7%) and (96.4%), respectively, was reported among *S. aureus*, while for CoNS, the reported susceptibility was 99.7% and 99.6% and for *Enterococcus* species, 99.5% and 85.9%, respectively [32].

The highest percentage of resistance among gram-positive organisms was exhibited by erythromycin, cotrimoxazole, and doxycycline, and the least resistance was reported for vancomycin. An Egyptian study reported that the highest resistance was observed for penicillin (89.5%), followed by erythromycin (83.98%), and then cefoxitin (76.52%). resistance to vancomycin was minimal (2.62%), and none of the isolates showed resistance to linezolid [25], while vancomycin had the best susceptibility profile to Staph aureus, CoNS, and *Enterococcus* species in this study. This was also reported in a study conducted in Saudi Arabia [27].

Another study conducted in Pakistan revealed an increasing resistance pattern of *Enterococcus* spp. for ampicillin, gentamicin, and ciprofloxacin from 2016 to 2019, and resistance to only ampicillin decreased after the COVID-19 pandemic, while for *S. aureus*, the authors reported an increase in resistance for Oxacillin and erythromycin from 2016 to 2019 and a decrease in resistance after the COVID-19 pandemic [5].

Our study included ciprofloxacin and levofloxacin; resistance to ciprofloxacin remained stable, while levofloxacin resistance increased in the last two years. While resistance to levofloxacin increased in the last 2 years, this is different from a study conducted in Egypt [24], which stated high resistance for ciprofloxacin and low resistance for levofloxacin. The wide use of these antibiotics in Egypt might explain this high rate of fluoroquinolone resistance. Another study conducted by Saini et al. [33] reported a lower susceptibility of gram-positive and gram-negative isolates for ciprofloxacin. In the current study, most of the gram-positive isolates were retrieved from blood samples (78%), where the most common gram-positive isolate retrieved from the blood samples was CoNS, which represents 59.4%. All respiratory samples revealed the growth of staph aureus, which represents 5%, while MRSA represents 141/158 (89%) of all respiratory samples.

This is in accordance with a study conducted in Romania by Golli et al. [34], which reported that CoNS was the most frequent pathogen implicated in blood stream infections during the pre- and post-COVID-19 pandemic era, while for respiratory samples including sputum and tracheal aspirates, followed by pus samples, *S. aureus* was most frequently isolated organism.

Different results were reported in India [34], where out of the reported 4428 gram-positive isolates retrieved over three years, Staph aureus (35.3%) was the commonly isolated pathogen, followed by *Enterococcus* spp. (32.1%), and the least was CoNS (25.7%). Most of the *S. aureus* isolates were isolated from skin and soft tissue infections (60.3%), followed by respiratory tract samples (18.2%), then blood stream infections (13%). MRSA represents 33.6% of the isolated organisms. These authors also concluded that high resistance was observed among gram-positive isolates for commonly used antibiotics, such as erythromycin, ciprofloxacin, and levofloxacin.

Collaboration between clinicians and microbiologists is crucial to accurately interpret diagnostic results [35]. Misidentifying CoNS as a true pathogen when it is merely a contaminant can lead to unnecessary antibiotic treatments, increased healthcare costs, and heightened antibiotic resistance risks [36]. To differentiate true infections from colonization, key diagnostic indicators include the following: a repeated isolation of the same strain from an infected site and the presence of clinical symptoms consistent with blood stream infections accompanied by positive blood cultures [37,38,39].

In this study, an incidence of drug resistance was observed towards erythromycin, cotrimoxazole, and doxycycline, and this may be attributed to different factors, such as different practices in Egypt, where some patients take a variety of oral antibiotics by self-medication as over-the-counter antibiotics that are typically taken in incorrect dosages and for insufficient lengths of time. It is mandatory to keep in mind that the data provided in this study only offers an overview of the current situation in the hospital that is the subject of this investigation. This highlights the importance of adding an action plan to optimize the use of antibiotics and control the emergence of resistance.

Our results confirm previous findings that vancomycin remains the most effective antibiotic against *Staphylococcus aureus*, CoNS, and *Enterococcus* species, whereas erythromycin displayed low efficacy against MRSA, CoNS, and *Enterococcus* species [40,41]. However, we observed a lower susceptibility rate of vancomycin against *Enterococcus* species, possibly due to antibiotic selection pressure and selective reporting of susceptibility testing.

The discrepancy between the results in this study and other studies regarding the frequency of isolated pathogens could be attributed to different factors, such as health practices, environmental conditions, patient conditions, personal hygiene, the number of patients involved in each study, and infection control procedures.

Although the COVID-19 pandemic had adverse effects on healthcare systems and led to a rise in antimicrobial resistance in certain areas, this study highlights the critical importance of implementing robust antimicrobial stewardship programs, enhancing infection control, and continuously investing in AMR research and surveillance.

Significant differences in antibiogram results across different healthcare facilities and regions may indicate differences in patient populations, antimicrobial usage patterns, or improper implementation of hospital infection prevention and control that could be further investigated.

### Limitations

This study had a few limitations, including its retrospective design and the risk of misclassification and selection biases. For instance, even though the laboratories follow the highest standards, there may be a possibility that some isolates had some contaminants. Furthermore, since the hospital in this study is an important pediatric care hospital, they receive more complicated cases that may be caused by resistant pathogens which may not indicate the antibiotic susceptibility trend and microbiology of the general population. Nevertheless, our study’s findings will add to local and global data on antimicrobial susceptibility, especially with highly threatening infections.

## 5. Conclusions

This study highlights the critical role of systematic data collection and analysis in monitoring antimicrobial resistance. Nationwide surveillance is essential to provide policymakers, antimicrobial stewardship committees, infection control specialists, microbiologists, and epidemiologists with the necessary information to develop effective intervention strategies.

While our findings indicate an overall increase in gram-positive bacterial susceptibility to certain antibiotics over the past five years, the persistent resistance of *Staphylococcus aureus* to erythromycin, MRSA to gentamicin and erythromycin, and CoNS and *Enterococcus* species to erythromycin and the rising resistance of *Enterococcus* species to vancomycin remain significant concerns. Addressing these threats requires robust antimicrobial stewardship programs to curb further resistance development and ensure effective treatment options remain available.

## Figures and Tables

**Table 1:** The prevalence of gram-positive pathogens in the pediatric hospital over 5 years.

Organism	2018 No. (%)	2019 No. (%)	2020 No. (%)	2021 No. (%)	2022 No. (%)	*p* Value
CONS no. 2054	320 (15.58%)	372 (18.11%)	405 (19.72%)	509 (24.78%)	448 (21.81%)	≤0.001
*S. aureus* no. 869 (MSSA and MRSA)	183 (21.06%)	177 (20.37%)	207 (23.82%)	160 (18.41%)	142 (16.34%)	≤0.001
*Enterococci* no. 300	73 (24.33%)	69 (23%)	48 (16%)	47 (15.67%)	63 (21%)	0.001
Total no. of gram-positive pathogens no. 3223	576 (17.87%)	618 (19.17%)	660 (20.48%)	716 (22.22%)	653 (20.26%)	≤0.001

**Table 2:** The prevalence of superbugs in the pediatric hospital over 5 years.

MDR Organism No. (%)	2018 No. (%)	2019 No. (%)	2020 No. (%)	2021 No. (%)	2022 No. (%)	*p* Value
MRSA 716/869 (82.5%)	166/183 (90.7%)	153/177 (86.4%)	172/207 (83%)	137/160 (85.6%)	88/142 (62%)	≤0.001
VRE 18/300 (6%)	5/73 (6.8%)	1/69 (1.4%)	8/48 (16.7%)	3/47 (6.4%)	1/63 (1.6%)	0.006

**Table 3:** Susceptibility profile of gram-positive bacteria over 5 years.

Antibiotic	2018	2019	2020	2021	2022	*p* Value
CIP Ciprofloxacin	234/505 (46.33%)	276/580 (47.59%)	289/593 (48.74%)	290/587 (49.4%)	510/1179 (43.26%)	0.077
LEV Levofloxacin	204/367 (55.59%)	321/537 (59.78%)	312/435 (71.72%)	280/494 (56.68%)	369/771 (47.86%)	≤0.001
DA Clindamycin	346/455 (76.04%)	262/488 (53.69%)	301/584 (51.54%)	265/526 (50.38%)	464/971 (47.79%)	≤0.001
SXT Cotrimoxazole	197/445 (44.27%)	192/455 (42.2%)	241/548 (43.98%)	201/485 (41.44%)	666/1119 (59.52%)	≤0.001
DXT Doxycycline	270/539 (50.09%)	384/564 (68.09%)	463/629 (73.61%)	355/516 (68.8%)	454/711 (63.85%)	≤0.001
E Erythromycin	140/532 (26.32%)	129/525 (24.57%)	139/633 (21.96%)	145/584 (24.83%)	203/930 (21.83%)	0.239
GM low-potency Gentamycin	149/452 (32.96%)	139/472 (29.45%)	280/583 (48.03%)	184/462 (39.83%)	389/963 (40.39%)	≤0.001
GM high-potency Gentamycin	42/66 (64%)	31/42 (73.8%)	19/33 (57.6%)	12/21 (57.1%)	72/297 (74.2%)	≤0.001
VA Vancomycin	564/573 (98.43%)	596/598 (99.67%)	642/654 (98.17%)	584/596 (97.99%)	1287/1317 (97.72%)	0.049

**Table 4:** Antimicrobial susceptibility profile of. *Staphylococcus aureus* over 5 years.

Antibiotic	2018	2019	2020	2021	2022	*p* Value
CIP	80/160 (50%)	90/160 (56.3%)	88/181 (48.6%)	84/144 (58.3%)	184/333 (55.3%)	0.315
LEV	55/100 (55%)	94/151 (62.3%)	93/140 (66.4%)	83/124 (66.9%)	111/180 (61.7%)	0.353
DA	91/164 (55%)	94/152 (61.8%)	108/204 (52.9%)	84/144 (58.3%)	196/336 (58.3%)	0.510
SXT	125/165 (76%)	99/148 (66.9%)	114/191 (59.7%)	85/133 (63.9%)	307/388 (79.1%)	≤0.001
DXT	67/170 (39%)	96/151 (63.6%)	135/201 (67.2%)	81/141 (57.4%)	132/230 (57.4%)	≤0.001
E	74/175 (42%)	76/147 (51.7%)	76/203 (37.4%)	65/145 (44.8%)	130/275 (47.3%)	0.078
GM low potency	46/159 (29%)	28/178 (15.7%)	74/194 (38.1%)	26/118 (22%)	109/340 (32.1%)	≤0.001
VA	181/181 (100%)	167/167 (100%)	206/206 (100%)	153/153 (100%)	391/391 (100%)	-

**Table 5:** Antimicrobial susceptibility profile of MRSA over 5 years.

Antibiotic	2018	2019	2020	2021	2022	*p* Value
CIP	67/145 (46%)	71/138 (51.4%)	63/151 (41.7%)	67/125 (53.6%)	130/258 (50.4%)	0.266
LEV	53/98 (54%)	72/127 (56.7%)	67/112 (59.8%)	68/107 (63.6%)	69/124 (55.6%)	0.646
DA	78/147 (53%)	73/127 (57.5%)	83/169 (49.1%)	68/125 (54.4%)	116/211 (55%)	0.672
SXT	110/150 (73%)	84/126 (66.7%)	89/153 (58.2%)	64/112 (57.1%)	235/303 (77.6%)	≤0.001
DXT	59/153 (38.5%)	80/126 (63.5%)	109/166 (65.7%)	69/125 (55.2%)	112/198 (56.6%)	≤0.001
E	62/157 (39%)	58/124 (46.8%)	55/165 (33.3%)	54/126 (42.9%)	59/156 (37.8%)	0.189
GM low potency	38/148 (62%)	12/157 (7.6%)	45/158 (28.5%)	18/103 (17.5%)	69/267 (25.8%)	≤0.001
VA	165/165 (100%)	144/144 (100%)	170/170 (100%)	131/131 (100%)	310/310 (100%)	-

**Table 6:** Antimicrobial susceptibility of CoNS profile over 5 years.

Antibiotic	2018	2019	2020	2021	2022	*p* Value
CIP	134/287 (47%)	159/352 (45.2%)	185/356 (52%)	198/399 (49.6%)	277/675 (41%)	0.007
LEV	118/205 (57.5%)	196/326 (60.1%)	206/261 (78.9%)	185/338 (54.7%)	218/498 (43.8%)	≤0.001
DA	149/291 (51%)	168/336 (50%)	193/380 (50.8%)	181/382 (47.4%)	268/635 (42.2%)	0.024
SXT	72/280 (26%)	93/307 (30.3%)	127/357 (35.6%)	116/352 (33%)	359/731 (49.1%)	≤0.001
DXT	182/308 (59%)	263/348 (75.6%)	298/384 (77.6%)	260/334 (77.8%)	288/391 (73.7%)	≤0.001
E	53/301 (18%)	51/316 (16.1%)	59/384 (15.4%)	75/395 (19%)	67/571 (11.7%)	0.027
GM low potency	103/293 (35%)	111/294 (37.8%)	206/389 (53%)	158/344 (45.9%)	280/623 (44.9%)	≤0.001
VA	323/323 (100%)	366/366 (100%)	400/400 (100%)	402/402 (100%)	740/740 (100%)	-

**Table 7:** Antimicrobial susceptibility profile of *Enterococci* over 5 years.

Antibiotic	2018	2019	2020	2021	2022	*p* Value
AMC	30/65 (46%)	33/69 (47.8%)	10/40 (25%)	4/22 (18.2%)	68/141 (48.2%)	0.010
CIP	20/58 (37%)	27/68 (39.7%)	16/56 (28.6%)	8/44 (18.2%)	49/171 (28.7%)	0.153
LEV	31/62 (50%)	31/60 (51.7%)	13/34 (38.2%)	12/32 (37.5%)	40/93 (43%)	0.525
DXT	21/61 (34%)	25/65 (38.5%)	30/44 (68.2%)	14/41 (34.1%)	34/90 (37.8%)	0.003
E	13/56 (32%)	2/62 (3.2%)	4/46 (8.7%)	5/44 (11.4%)	6/84 (7.1%)	0.006
GM high potency	42/66 (64%)	31/42 (73.8%)	19/33 (57.6%)	12/21 (57.1%)	72/297 (74.2%)	≤0.001
VA	60/69 (87%)	63/65 (96.9%)	36/48 (75%)	29/41 (70.7%)	160/186 (86%)	0.001

## Data Availability

The datasets generated during and/or analyzed during the current study are available from the corresponding author on reasonable request.

## Supplementary Materials

The following supporting information can be downloaded at: https://www.mdpi.com/article/10.3390/medicina61061089/s1, Table S1: Prevalence and distribution of staph aureus in different specimens over 5 years; Table S2: Prevalence and distribution of MRSA in different specimens over 5 years; Table S3: Prevalence and distribution of CONS in different specimens over 5 years; Table S4: Prevalence and distribution of Enterococci in different specimens over 5 years.

## Abbreviations

The following abbreviations are used in this manuscript:

Abbreviation	Details
MRSA	Methicillin-resistant *Staphylococcus aureus*
CoNS	Coagulase-negative *Staphylococcus*
AMR	Antimicrobial resistance
AMS	Antimicrobial stewardship
HAIs	Healthcare-associated infections
CSF	Cerebrospinal fluid
CLSI	Clinical Laboratory Standard Institute
EUCAST	European Committee on Antimicrobial Susceptibility Testing
MIC	Minimum Inhibitory Concentration
VRSA	Vancomycin-resistant *Staphylococcus aureus*
MDROs	Multidrug-resistant organisms
VRE	Vancomycin-resistant *Enterococcus*
