# Supplementary material for: A Retrospective Study on the Prevalence and Antimicrobial Susceptibility of Gram-Positive Cocci in a Pediatric Department: A Single-Center Report from Egypt
Source: Medicina (Kaunas). 2025 Jun 14;61(6):1089. doi: 10.3390/medicina61061089 (PMC12195014; doi:10.3390/medicina61061089)
Supplement: Supplementary file 1 [file medicina-61-01089-s001.zip › medicina-3627355-supplementary.pdf]

The prevalence and distribution of digfferent gram positive from different clinical samples presented in tables (Tables 1–4)

Table S1. Prevalence and distribution of staph aureus in different specimens over 5 years

Specimen	2018	2019	2020	2021	2022	P value
**Blood 482**	82 (17.01%)	89 (18.46%)	130 (26.97%)	98 (20.33%)	83 (17.22%)	**0.002**
**Urine 23**	4 (17.39%)	3 (13.04%)	7 (30.43%)	5 (21.74%)	4 (17.39%)	0.850
**Respiratory 158**	49 (31.01%)	30 (18.99%)	32 (20.25%)	19 (12.03%)	28 (17.72%)	**0.005**
**Pus & wound 196**	45 (22.96%)	54 (27.55%)	36 (18.37%)	35 (17.86%)	26 (13.27%)	**0.021**
**CSF 8**	2 (25%)	1 (12.5%)	1 (12.5%)	3 (37.5%)	1 (12.5%)	0.661
**Pleural 2**	1 (50%)	0 (0%)	1 (50%)	0 (0%)	0 (0%)	0.648

Table S2. Prevalence and distribution of MRSA in different specimens over 5 years.

Specimen	2018	2019	2020	2021	2022	P value
**Blood 374**	75 (20.05%)	72 (19.25%)	107 (28.61%)	81 (21.66%)	39 (10.43%)	**0.002**
**Urine 17**	3 (17.65%)	2 (11.76%)	7 (41.18%)	3 (17.65%)	2 (11.76%)	0.535
**Respiratory 141**	45 (31.91%)	28 (19.86%)	26 (18.44%)	18 (12.77%)	24 (17.02%)	**0.004**
**Pus & wound 175**	41 (23.43%)	50 (28.57%)	30 (17.14%)	32 (18.29%)	22 (12.57%)	**0.036**
**CSF 7**	1 (14.29%)	1 (14.29%)	1 (14.29%)	3 (42.86%)	1 (14.29%)	0.594
**Pleural 2**	1 (50%)	0 (0%)	1 (50%)	0 (0%)	0 (0%)	0.691

Table S3. Prevalence and distribution of CONS in different specimens over 5 years.

Specimen	2018	2019	2020	2021	2022	P value
**Blood 1917**	296 (15.44%)	349 (18.21%)	378 (19.72%)	481 (25.09%)	413 (21.54%)	0.635
**Urine 24**	6 (25%)	6 (25%)	5 (20.83%)	4 (16.67%)	3 (12.5%)	0.456
**Pus & wound 85**	17 (20%)	16 (18.82%)	16 (18.82%)	13 (15.29%)	23 (27.06%)	0.239
**CSF 26**	1 (3.85%)	1 (3.85%)	6 (23.08%)	10 (38.46%)	8 (30.77%)	0.078
**Pleural 2**	0 (0%)	0 (0%)	0 (0%)	1 (50%)	1 (50%)	0.679

Table S4. Prevalence and distribution of Enterococci in different specimens over 5 years

Specimen	2018	2019	2020	2021	2022	P value
**Blood**<br>**116**	24<br>(20.69%)	26<br>(22.41%)	19<br>(16.38%)	19<br>(16.38%)	28<br>(24.14%)	0.731
**Urine**<br>**127**	26<br>(20.47%)	34<br>(26.77%)	22<br>(17.32%)	23<br>(18.11%)	22<br>(17.32%)	0.266
**Pus & wound**<br>**56**	23<br>(41.07%)	9<br>(16.07%)	7<br>(12.5%)	5<br>(8.93%)	12<br>(21.43%)	**0.018**
**CSF**<br>**1**	0<br>(0%)	0<br>(0%)	0<br>(0%)	0<br>(0%)	1<br>(100%)	0.437
